# Gastrointestinal problem among Indian adults: Evidence from longitudinal aging study in India 2017–18

**DOI:** 10.3389/fpubh.2022.911354

**Published:** 2022-09-26

**Authors:** Sameer Dawoodi, Inshiya Dawoodi, Priyanka Dixit

**Affiliations:** ^1^Bridgeport Hospital, Yale New Haven Health, Bridgeport, CT, United States; ^2^Griffin Hospital, Derby, CT, United States; ^3^School of Health Systems Studies, Tata Institute of Social Sciences, Deonar, India

**Keywords:** gastrointestinal problems, adults, longitudinal aging study in India-2017–18, India, aging

## Abstract

**Introduction:**

Diseases and illnesses of the gastrointestinal system (GIS) have grown in the last decade due to considerable lifestyle changes. People with gastrointestinal (GI) diseases have a high prevalence of depression, stress, anxiety, and impaired central nervous system functioning. Therefore, this study aims to explore the factors associated with the self-reported gastrointestinal problems among the Indian elderly and to explore the relationship between non-communicable diseases (NCDs), such as hypertension, heart diseases, diabetes, and neurological or psychiatric and gastrointestinal disorder.

**Methods:**

This study uses data from the Longitudinal Aging Study in India (LASI), a population-based national survey, conducted during 2017–2018 with a representative sample of 72,250 individuals. Descriptive statistics were used to provide the frequency distribution of sociodemographic and economic profiles of adults. Bivariate analysis was used to understand the percentage distribution of adults suffering from gastrointestinal problems by their background characteristics. Binary logistic regression was used to determine the factors associated with gastrointestinal problems. In the binary logistic regression analysis, a systematic model building procedure was adopted.

**Results:**

The overall prevalence of self-reported gastrointestinal problems was 18%, with significant variations among regions, and it substantially increased with the increasing age of men. Hypertension and neurological problems have significant individual effects on gastrointestinal problems. Prevalence was higher in those who suffered from neurological or psychiatric problems (27%) than in those who suffered from hypertension (22%) and heart disease (23%). Adults from the age group 45–54 (1.11, *p* < 0.01) and 55–64 (1.09, *p* < 0.01) years were significantly more likely to have gastrointestinal problems compared with the <44 years age group. Former and current smokers and adults with the habits of chewing tobacco were significantly more likely to report gastrointestinal problems than their counterparts. Moreover, the increasing economic status significantly and positively increased the likelihood of having self-reported gastrointestinal problems among adults.

**Conclusion:**

Aging-related gastrointestinal problems are physiological or pathological and more prevalent in the elderly population aged 64 years and above. Hence, policies and interventions have to be made age-specific. Gastrointestinal problems among older adults are acquiring greater importance in clinical practices to plan effective treatment, administration of gastrointestinal drugs, the early screening of gastrointestinal diseases. Given the policy focus through Health and Wellness centers for accessible NCD care, it is important that gastro-intestinal illnesses receive more focus and systemic support.

## Introduction

Aging is characterized by a steady loss of physiological integrity, which results in reduced function and an increased risk of mortality. This degradation is a key risk factor for the majority of human illnesses, such as cancer, diabetes, cardiovascular disease, and neurological diseases (1, 2). All functions of the gastrointestinal system (GIS) are affected by aging, such as motility, enzyme and hormone release, digesting, and absorption. In addition, the GIS is involved in the absorption and metabolism of medications, and it is frequently impacted by adverse effects (2). In the absence of organic illness, functional gastrointestinal disorders (FGIDs) are diseases with chronic or recurrent symptoms related to the gastrointestinal (GI) tract that can be diagnosed by standard examinations (3). FGIDs do not lead to an increase in mortality, but they do cause considerable morbidity in the terms of lowering the quality of life and increasing healthcare utilization (4–8).

A number of population-based studies documented that FGIDs were more prevalent among women than men and were associated with lower quality of life and more frequent visits to the doctor (9, 10). A large-scale multinational study based on internet surveys has shown that >40% of people worldwide suffer from gastrointestinal complications, such as diarrhea, constipation, or irritable bowel syndrome (IBS), with a prevalence of 4.7, 11.7, and 4.1%, respectively (9). Increasing age, on the other hand, is linked to an increase in gastrointestinal motility problems in both men and women, such as constipation, diarrhea, or incontinence (11).

Due to considerable lifestyle changes, diseases and illnesses of the gastrointestinal system have grown in the last decade. Various studies have shown that people with gastrointestinal diseases have a high prevalence of depression, stress, anxiety, and impaired central nervous system functioning (9, 12–14).

Limited studies have been conducted to investigate the determinants associated with gastrointestinal disorders among older adults in India. The study aims to explore the factors associated with self-reported gastrointestinal problems among the Indian elderly using the recently released Longitudinal Aging Study in India (LASI, 2017–18). Moreover, the study explores the relationship between non-communicable diseases (NCDs), such as hypertension, heart diseases, diabetes, and neurological or psychiatric and gastrointestinal disorders.

## Materials and methods

### Data source and study population

We used unit data from large-scale population-based surveys, namely, the Longitudinal Aging Study in India (LASI), wave 1, conducted during 2017–18. A brief description of the data structure of the surveys is given below.

The LASI is a nationally representative study on the health, economic, and social wellbeing of older adults (45+ years) and their spouses in India, covering extensive biomarkers and self-reported health measures. It is a collaborative study of the International Institute for Population Sciences (IIPS), Mumbai, India, the Harvard T.H. Chan School of Public Health, and the University of Southern California, the United States. It has the distinction of being the largest-ever study, worldwide, with a representative sample of 72,250 individuals and 42,949 age-eligible households across all states and union territories of India except Sikkim. The age-eligible households included those households who had at least one member aged 45+ years. The LASI used a multistage stratified area probability cluster sampling design in the selection of the sample households. It provided comprehensive information on household economic wellbeing, work and employment, retirement and pension, chronic health conditions, functional health, mental health, extensive biomarkers, healthcare utilization, and health insurance of older adults in India.

### Dependent variable

The dependent variable for this study is the self-reported gastrointestinal problems among the elderly (45+ years), coded as “0” for no and “1” for yes. In LASI, gastrointestinal problems include gastroesophageal reflux disease, constipation, indigestion, piles, and peptic ulcer.

### Independent variable

A number of independent variables have been used in this study. Individual factors, such as the age groups (<44, 45–54, 55–64, 65–74, and 75+ years), education level (no education, primary, secondary, and higher), currently working (never worked, currently working, and not currently working), marital status (currently married, divorced/others, and widowed), the presence of non-communicable diseases, such as diabetes (yes, no), stroke (yes, no), arthritis (yes, no), hypertension (yes, no), heart diseases (yes, no), neurological (yes, no) or psychiatric (yes, no), difficulty in Activities of Daily Living (ADL) (yes, no), and difficulty in Instrumental Activities of Daily Living (IADL) (yes, no) were included in the analysis. In addition, lifestyle factors, such as moderate activities (inactive, active), vigorous activities (inactive, active), smoking status (yes, no), chewing status (yes, no), and drinking status (yes, no) were included in the analysis. LASI collected information from households about the last 7 days of their spending on food (a reference period of 7 days) and non-food items (reference periods of 30 and 365 days). After standardizing the food and non-food expenditure to a 30-day reference period, the monthly per capita consumption expenditure (MPCE) was computed and used as the summary measure of consumption: poorest, poorer, middle, richer, and richest. Apart from these independent variables, the study also included the household factors, such as religion (Hindu, Muslim, Christian, and Others), castes [Scheduled castes (SCs), Scheduled Tribes (STs), Other Backward Classes (OBCs), and Others], place of residence (rural or urban), and region (north, central, east, northeast, west, and central) have been included in the analysis.

### Methods

Descriptive, bivariate, and multivariable statistical techniques have been used in this study. Descriptive statistics were used to provide the frequency distribution of sociodemographic and economic profiles of adults in India. A bivariate analysis was used to understand the percentage distribution of adults suffering from gastrointestinal problems by their background characteristics in India.

A binary logistic regression was used to determine the factors associated with gastrointestinal problems. The basic form of the logistic regression model, which yields the probability of occurring of an event, can be depicted as:


(1) $$\begin{array}{l} log_{e}\left[P\left(Y_{i}=1|X_{i}\right)/1-P\left(Y_{i}=1|X_{i}\right)\right]=loge[\pi |1-\pi ]=\alpha +\beta_{1}X_{i1},\ldots \ldots \ldots \beta_{k}X_{ik} \end{array}$$

Where *Yi* is the binary response variable and *X*_*i*_ is the set of explanatory variables. The socio-demographic variables (Xi) used are age, sex, education, place of residence, caste, household size, and states. *β*_1_, *β*_2, ……_ *β*_*k*_are the coefficients of the *X*_*i*_ variables.

In the binary logistic regression analysis, a systematic model-building procedure was adopted. Altogether, two models were estimated. Model 1 included the individual factors, lifestyle factors, and household factors along with non-communicable diseases, whereas model 2 included the interaction of hypertension and neurological disorder in addition to the variables included in model 1 to understand the independent effect of the interaction of hypertension and neurological problems on gastrointestinal disorders after controlling the potential confounding variables.

## Results

Table 1 presents the socio-demographic and economic profile of adults in India. The majority of the adults (32%) reported their age as 45–54 years. A substantial proportion (10%) of adults belonged to the age group of 75 years and above and only 9% of them belonged to the age group of 44 years or less. In India, the prevalence of gastrointestinal problems was 18%. Approximately half of the adults (50%) were not educated and 23% of them received primary education only. About 17% of adults had attained the secondary level of education, whereas only 10% of the adults attained a higher level of education. Nearly half (46%) of the adults were currently working, however, 26% of the adults were not currently working and the remaining 28% had never worked. In the sample, 78% of the adults were currently married and a substantial proportion (22%) of the respondents were widowed. Wealth Index analysis portrays that an almost equal sample comprised of at each level of MPCE quintile varies from 21% in poorest, poorer, and middle to 18% in richest. The majority of the respondents belonged to the Hindu religion (82%), followed by Muslims (12%), and the remaining were Christians (3%) and other religions [Sikh, Buddhist, etc. (3%)]. A significant proportion of the respondent comprised Other Backward Classes (OBCs) 46%, while Schedule Castes (SCs) constituted 19% and Schedule Tribes (STs) 9% of the proportions of the sample. Findings depict that 68% of adults belonged to rural areas. In addition, regional variation was noticed in the sample of adults. A majority of women belonged to the Southern (24%), followed by Eastern (23%), Central (20%), Western (17%), Northern (12.0%), and North-eastern (4%) part of the country.

**Table 1:** Socio-demographic and economic profile of adults in India, LASI-2017–18.

**Background** ** characteristics**	**%**	**Sample**
**Age groups**
<44	6,688	8.69
45–54	24,094	31.91
55–64	20,136	27.25
65–74	14,583	21.72
75+	6,749	10.43
**Education level**
No education	33,211	49.50
Primary	17,736	23.21
Secondary	13,949	16.91
Higher	7,354	10.38
**Currently working**
Never worked	21,289	27.57
Currently working	32,990	46.28
Not currently working	17,951	26.14
**Marital status**
Currently married	55,396	75.60
Widowed	14,593	21.66
D/S/D/Others^a^	2,257	2.73
**MPCE quintile**
Poorest	14,158	20.70
Poorer	14,530	21.22
Middle	14,537	20.47
Richer	14,686	19.59
Richest	14,339	18.03
**Religion**
Hindu	52,973	81.92
Muslim	8,667	11.67
Christian	7,215	2.97
Others^$^	3,390	3.44
**Caste**
Scheduled caste	12,046	19.15
Scheduled tribe	12,509	8.53
OBC^#^	27,184	45.50
Others	20,511	26.82
**Place of residence**
Rural	46,534	68.20
Urban	25,716	31.80
**Region**
North	12,970	12.01
Central	9,536	20.10
East	12,834	23.40
Northeast	9,676	3.64
West	9,822	16.48
South	17,412	24.38
Total	72,250	100

a Divorced, separated, and deserted;

$ Includes Sikh, Buddhist/neo-Buddhist, Jain, Parsi/Zoroastrian and others;

# Other Backward Classes.

Table 2 presents the percentage of adults suffering from gastrointestinal problems by their background characteristics in India. The overall prevalence of self-reported gastrointestinal problems was 18%. However, the prevalence of self-reported gastrointestinal problems was 1% higher among men (18%) than women (17%). The prevalence of self-reported gastrointestinal problems has substantially increased with the increasing age of men; however, men (20%) and women (20%) of 75+ years of age are at a high risk of suffering gastrointestinal problems. The prevalence of reported gastrointestinal problems was relatively lesser among those who had a higher level of schooling for both men (16%) and women (10%). An almost equal proportion of never worked and not currently working adults (19%) had reported gastrointestinal problems. Self-reported gastrointestinal problems were comparatively lesser among D/S/D/Others adults (12%) than currently married (18%) and widowed adults (18%). It was observed that the prevalence of self-reported gastrointestinal problems was higher among adults who had stroke (23% yes vs. 17% no), arthritis (23% yes vs. 17% no), difficulty in ADL (23 vs. 17%), and difficulty in IADL (21 vs. 17%).

**Table 2:** Percentage of adults suffering from GI by their background characteristics in India, LASI 2017–18.

	**Self-reported gastrointestinal problem**					
**Background characteristics**	**Overall** **(*****n*** **=** **72,037)**	***p*** **-value**	**Men (*****n*** **=** **30,460)**	***p*** **-value**	**Women** **(*****n*** **=** **41,577)**	***p*** **-value**
**Individual factors**	**%**		**%**		**%**	
**Age groups**						
<44	15.3	*p* = 0.000	9.37	*p* = 0.063	15.33	*p* = 0.000
45-54	16.73		17.02		16.50	
55-64	18.62		18.46		18.75	
65-74	18.40		19.25		17.63	
75+	20.06		20.46		19.69	
**Education level**						
No education	17.70	*p* = 0.000	17.31	*p* = 0.000	17.86	*p* = 0.000
Primary	19.41		19.90		18.90	
Secondary	18.78		19.86		17.37	
Higher	13.36		15.79		9.56	
**Currently working**						
Never worked	18.82	*p* = 0.000	15.37	*p* = 0.00	18.99	*p* = 0.000
Currently working	16.67		17.86		14.96	
Not currently working	18.83		19.79		17.84	
**Marital status**						
Currently married	18.15	*p* = 0.000	18.54	*p* = 0.000	17.79	*p* = 0.007
Widowed	17.45		19.04		17.06	
D/S/D/Others^a^	11.88		11.48		12.18	
**Morbidities**						
**Diabetes**						
No	17.78	*p* = 0.000	18.32	*p* = 0.009	17.40	*p* = 0.000
Yes	18.18		18.88		17.61	
**Stroke**						
No	17.73	*p* = 0.000	18.24	*p* = 0.001	17.37	*p* = 0.021
Yes	23.06		24.14		21.54	
**Arthritis**						
No	17.35	*p* = 0.000	17.83	*p* = 0.000	17.00	*p* = 0.000
Yes	22.68		25.52		21.20	
**Difficulty in ADL** ^**b**^						
No	16.81	*p* = 0.000	17.62	*p* = 0.000	16.21	*p* = 0.000
Yes	23.12		22.55		23.48	
**Difficulty in IADL** ^**c**^						
No	16.27	*p* = 0.000	16.90	*p* = 0.000	15.72	*p* = 0.000
Yes	20.52		21.95		19.82	
**Lifestyle factors**						
**Moderate activities**						
Inactive	16.83	*p* = 0.000	17.20	*p* = 0.000	16.37	*p* = 0.000
Active	18.40		19.43		17.85	
**Vigorous activities**						
Inactive	18.2	*p* = 0.000	18.2	*p* = 0.432	18.20	*p* = 0.000
Active	17.06		18.64		15.18	
**Smoking status**						
Never	17.31	*p* = 0.000	17.51	*p* = 0.000	17.21	*p* = 0.000
Former	25.00		24.20		30.44	
Current	19.38		18.96		22.60	
**Chewing tobacco**						
Never	16.51	*p* = 0.000	16.64	*p* = 0.000	16.43	*p* = 0.000
Former	24.74		23.21		27.51	
Current	22.23		22.01		22.54	
**Drinking status**						
No	17.55	*p* = 0.001	17.69	*p* = 0.000	17.48	*p* = 0.045
Yes	19.56		20.00		15.76	
**Household factors**						
**MPCE quintile**						
Poorest	15.63	*p* = 0.000	16.59	*p* = 0.000	14.95	*p* = 0.000
Poorer	18.21		18.73		17.83	
Middle	17.84		18.30		17.51	
Richer	19.21		19.79		18.80	
Richest	18.38		18.62		18.20	
**Religion**						
Hindu	17.60	*p* = 0.000	18.17	*p* = 0.00	17.18	*p* = 0.000
Muslim	19.37		19.60		19.21	
Christian	17.35		18.50		16.67	
Others^$^	18.55		19.67		17.69	
**Caste**						
Scheduled caste	18.98	*p* = 0.000	20.15	*p* = 0.000	18.16	*p* = 0.000
Scheduled tribe	14.82		16.32		13.75	
OBC^#^	15.95		16.61		15.46	
Others	21.08		20.87		21.24	
**Place of residence**						
Rural	19.38	*p* = 0.000	19.98	*p* = 0.000	18.93	*p* = 0.000
Urban	14.48		14.69		14.33	
**Region**						
North	22.74	*p* = 0.000	20.85	*p* = 0.000	24.13	*p* = 0.000
Central	16.91		17.30		16.58	
East	26.29		25.88		26.59	
Northeast	23.11		21.82		24.00	
West	12.57		14.79		11.06	
South	10.73		12.16		9.83	
**NCD**						
Hypertension	22.11	*p* = 0.000	21.82	*p* = 0.000	22.28	*p* = 0.000
Heart disease	22.57	*p* = 0.000	22.02	*p* = 0.000	23.09	*p* = 0.000
Neurological or psychiatric problems	26.99	*p* = 0.000	25.17	*p* = 0.000	28.37	*p* = 0.000
Total	17.83		18.38		17.42	

a Divorced, separated, and deserted.

b Activities of daily living includes dressing, walking across a room, bathing, eating difficulties, getting in or out of bed and toilet use (any one or more).

c Instrumental Activities of Daily Living (IADL) includes preparing a hot meal, shopping for groceries, making telephone calls, taking medications, doing work around the house or garden, managing money and getting around or finding address in unfamiliar place (any one or more).

# Other Backward Classes.

$ Includes Sikh, Buddhist/neo-Buddhist, Jain, Parsi/Zoroastrian and others.

By considering the lifestyle factors, it was found that adults who were active in moderate activities were more likely (18%) to report gastrointestinal problems than those who were inactive (17%). Among adults who were inactive in vigorous activities, the self-reported prevalence of gastrointestinal problems was a little higher than those who were active. Light and moderate exercises are well-tolerated and can benefit patients with inflammatory bowel disease and liver disease but severe exhaustive exercises, however, inhibits gastric emptying, interferes with gastrointestinal absorption, and causes many gastrointestinal symptoms, most notably gastrointestinal bleeding (15). The self-reported gastrointestinal problem was higher among those adults who were formerly involved in smoking and chewing tobacco than those who were currently involved and never involved in smoking and chewing tobacco. However, the prevalence of self-reported gastrointestinal problems was higher among women than men who were former and currently involved in smoking and chewing tobacco. Adults who were drinking reported more gastrointestinal problems (20%) than those who were not drinking (18%). While observing separately among men and women, it was found that the self-reported gastrointestinal problem was higher among men who were drinking (20% yes vs. 18% no), however, in the case of women it was higher among those who were not drinking (16% yes vs. 18% no).

There are no consistent changes in the prevalence of self-reported gastrointestinal problems among adults that have been observed with the increasing MPCE quintile. Additionally, men and women from the richer and richest wealth quintile were at a higher risk of gastrointestinal problems than their other counterparts. Furthermore, the prevalence of self-reported gastrointestinal problems was a little higher among women and men who belong to the other social group (21%), rather than the Scheduled Tribes (STs, 16% men and 14% women), Scheduled Castes (SCs, 20% men and 18% women), and Other Backward Classes (OBCs, 17% men and 16% women). The prevalence of self-reported gastrointestinal problems was higher among men and women from rural areas (20% men and 19% women) than urban area (15% men and 14% women). The regional variation in the prevalence of self-reported gastrointestinal problems was also noticeable. The eastern region shows the highest prevalence of gastrointestinal problems among adults (26%) followed by the northeast (23%) and northern (23%) regions in India, where women were found to possess a higher burden of gastrointestinal problems, compared with men. Differences in access to healthcare and cultural factors, such as help-seeking behavior, may contribute to differences in the prevalence of men and women (16). Women have been shown to be more sensitive to pressure from an inflated balloon placed in the esophagus (swallowing tube between the mouth and the stomach), small intestine, colon or large intestine, and rectum than men. Among adults who have suffered from neurological or psychiatric problems (27%), the prevalence of self-reported gastrointestinal problems was higher than those who suffered from hypertension (22%) and heart disease (23%), however, the burden was higher among women (28%) than among men (25%).

Table 3 presents the adjusted odds ratio (*AOR*) estimates for self-reported gastrointestinal problems among adults by their background characteristics in India. Two models are presented in Table 3, model 1 analyses the individual factors, lifestyle factors, and household factors, whereas model 2 analyses the interaction between two NCDs, which strongly affect gastrointestinal problems along with the factors, which analyzed in model 1. Results from model 1 reveal that adults from the age groups of 45–54 (1.11, *p* < 0.01) and 55–64 (1.09, *p* < 0.01) years were significantly more likely to have gastrointestinal problems compared with the <44 years age group. Women were 1.15 (*p* < 0.01) times significantly more likely to report gastrointestinal problems than men. The education of the respondents was found to be significant and was negatively associated with the self-reported gastrointestinal problem in India. As the education level of the respondents increases, the odds of having gastrointestinal problems decrease simultaneously.

**Table 3:** Adjusted odds ratio estimates for GI among adults by their background characteristics in India.

**Background** ** characteristics**	**Model-1 (Full model)**	**Model-2 (Full model with interaction between two NCD which strongly affect GI)**
	**AOR 95% CI**	**AOR 95% CI**
**Age groups**
<44		
45–54	1.109^***^ (1.03, 1.20)	1.109^***^ (1.03, 1.20)
55–64	1.089^**^ (1.00, 1.18)	1.088^**^ (1.00, 1.18)
65–74	0.988 (0.9, 1.08)	0.988 (0.9, 1.08)
75+	0.939 (0.84, 1.05)	0.938 (0.84, 1.04)
**Sex**
Male		
Female	1.151^***^ (1.08, 1.22)	1.151^***^ (1.08, 1.22)
**Education level**
No education		
Primary	1.173^***^ (1.11, 1.23)	1.173^***^ (1.11, 1.23)
Secondary	1.116^***^ (1.05, 1.19)	1.116^***^ (1.05, 1.19)
Higher	0.902^**^ (0.83, 0.98)	0.902^**^ (0.83, 0.98)
**Currently working**
Never worked		
Currently working	0.984 (0.93, 1.04)	0.984 (0.93, 1.04)
Not currently working	1.045 (0.99, 1.11)	1.045 (0.99, 1.11)
**Marital status**
Currently married		
Widowed	0.923^***^ (0.87, 0.98)	0.923^***^ (0.87, 0.98)
D/S/D/Others^a^	0.768^***^ (0.68, 0.87)	0.768^***^ (0.68, 0.87)
**Morbidities**
**Diabetes**		
No		
Yes		
**Stroke**	1.065^**^ (1.001, 1.13)	1.065^**^ (1, 1.13)
No		
Yes	0.955 (0.83, 1.10)	0.957 (0.83, 1.10)
**Arthritis**
No		
Yes	1.319^***^ (1.23, 1.41)	1.319^***^ (1.23, 1.41)
**Difficulty in ADL** ^**b**^
No		
Yes	1.294^***^ (1.22, 1.37)	1.294^***^ (1.22, 1.37)
**Difficulty in IADL** ^**c**^
No		
Yes	1.24^***^ (1.18, 1.30)	1.24^***^ (1.18, 1.3)
**Lifestyle factors**
**Moderate activities**
Inactive		
	**AOR 95% CI**	**AOR 95% CI**
Active	1.188^***^ (1.14, 1.24)	1.188^***^ (1.14, 1.24)
**Vigorous activities**
Inactive		
Active	0.917^***^ (0.87, 0.96)	0.917^***^ (0.87, 0.96)
**Smoking status**
Never		
Former	1.448^***^ (1.32, 1.59)	1.448^***^ (1.32, 1.59)
Current	1.253^***^ (1.17, 1.34)	1.253^***^ (1.17, 1.34)
**Chewing tobacco**
Never		
Former	1.572^***^ (1.40, 1.77)	1.572^***^ (1.4, 1.77)
Current	1.472^***^ (1.40, 1.55)	1.472^***^ (1.4, 1.55)
**Drinking status**
No		
Yes	1.054^*^ (0.99, 1.12)	1.054^*^ (0.99, 1.12)
**Household factors**
MPCE quintile		
Poorest		
Poorer	1.203^***^ (1.13, 1.28)	1.203^***^ (1.13, 1.28)
Middle	1.219^***^ (1.14, 1.30)	1.218^***^ (1.14, 1.30)
Richer	1.358^***^ (1.27, 1.45)	1.358^***^ (1.27, 1.45)
Richest	1.347^***^ (1.26, 1.44)	1.347^***^ (1.26, 1.44)
**Religion**
Hindu		
Muslim	0.971 (0.91, 1.03)	0.971 (0.91, 1.03)
Christian	1.05 (0.96, 1.14)	1.05 (0.96, 1.14)
Others^$^	0.894^**^ (0.81, 0.98)	0.894^**^ (0.81, 0.98)
**Caste**
Scheduled caste		
Scheduled tribe	0.712^***^ (0.66, 0.77)	0.712^***^ (0.66, 0.77)
OBC^#^	0.929^**^ (0.88, 0.98)	0.928^**^ (0.88, 0.98)
Others	0.969 (0.91, 1.03)	0.969 (0.91, 1.03)
**Place of residence**
Rural		
Urban	0.962^*^ (0.92, 1.01)	0.962^*^ (0.92, 1.01)
**Region**
North		
Central	0.756^***^ (0.7, 0.81)	0.756^***^ (0.7, 0.81)
East	1.169^***^ (1.1, 1.25)	1.169^***^ (1.1, 1.25)
Northeast	0.846^***^ (0.78, 0.92)	0.847^***^ (0.78, 0.92)
West	0.463^***^ (0.43, 0.50)	0.463^***^ (0.43, 0.50)
South	0.539^***^ (0.5, 0.58)	0.539^***^ (0.5, 0.58)
	**AOR 95% CI**	**AOR 95% CI**
**NCD**
Heart disease	1.168^***^ (1.06, 1.29)	1.169^***^ (1.06, 1.29)
Hypertension	1.476^***^ (1.41, 1.54)	1.481^***^ (1.42, 1.55)
Neurological or psychiatric problems	1.344^***^ (1.19, 1.52)	1.424^***^ (1.21, 1.68)
**Hypertension**^**#**^ **neurological**		
Yes^#^Yes		0.883 (0.70, 1.12)
Constant	0.135 (0.12, 0.15)	0.135 (0.12, 0.15)
Log likelihood	−32,651.132	−32,650.607

# Other Backward Classes.

$ Includes Sikh, Buddhist/neo-Buddhist, Jain, Parsi/Zoroastrian and others.

a Divorced, separated, and deserted.

b Activities of daily living includes dressing, walking across a room, bathing, eating difficulties, getting in or out of bed and toilet use (any one or more).

c Instrumental Activities of Daily Living (IADL) includes preparing a hot meal, shopping for groceries, making telephone calls, taking medications, doing work around the house or garden, managing money and getting around or finding address in unfamiliar place (any one or more).

* *p* < 0.05,

** *p* < 0.01,

*** *p* < 0.001.

The likelihood of being affected by gastrointestinal problems was found to be lower among adults who were widowed (*AOR* = 0.92, *p* < 0.01) and D/S/D/Others (*AOR* = 0.77, *p* < 0.01) as compared with the adults who were currently married. Furthermore, considering the association between morbidities and gastrointestinal problems, it was found that adults who suffered from diabetes (*AOR* = 1.07, *p* < 0.01), arthritis (*AOR* = 1.32, *p* < 0.01), difficulty in ADL (*AOR* = 1.29, *p* < 0.01), and difficulty in IADL (*AOR* = 1.24, *p* < 0.01) were significantly more likely to report gastrointestinal problems than their counterparts. Regarding the lifestyle factors, results reveal that adults who were active in moderate activities were significantly more likely (*AOR* = 1.18, *p* < 0.01) to report gastrointestinal problems than those who were inactive. In contrast, adults who were active in vigorous activities were significantly less likely (*AOR* = 0.92, *p* < 0.01) to report gastrointestinal problems than those who were inactive. Additionally, Table 3 reveals the effect of behavioral habits, such as smoking, chewing tobacco, and alcohol use, on the self-reported gastrointestinal problems. Furthermore, adults who were formerly and currently smoking and chewing tobacco were significantly more likely to report gastrointestinal problems than their counterparts. Moreover, the increasing economic status significantly and positively increased the likelihood of having self-reported gastrointestinal problems among adults. On the other hand, the risk of gastrointestinal problems was found to be significantly lower among adults who belong to the Scheduled Tribes (STs) (*AOR* = 0.71, *p* < 0.01) and Other Backward Classes (OBCs) (*AOR* = 0.93, *p* < 0.01) category of the social group. The regional variation in the prevalence of gastrointestinal problems is also noticeable. Except for the eastern region, all other regions were significantly less likely to report gastrointestinal problems than the northern region.

The likelihood of being affected by gastrointestinal problems was found to be higher among adults who were affected by NCDs, namely, heart disease, hypertension, and neurological or psychiatric problems. The odds of individual factors, lifestyle factors, and household factors were unchanged in model 2, whereas the association among being affected with hypertension, neurological problems, and self-reported gastrointestinal problems was less likely and insignificant. The insignificant interaction term clearly shows that hypertension and neurological problem have significant individuals effect on gastrointestinal problems.

## Discussion

In this study, we investigated the self-reported prevalence of gastrointestinal problems and the associated risk factors with an emphasis on non-communicable diseases, namely, heart disease, hypertension, and neurological disorders. The prevalence of self-reported gastrointestinal disorders was found to be ~18% in India. Whereas, the prevalence of gastroesophageal reflux disease (GERD) ranges from 2.5% to 7.1% in most population-based studies in Asia. No significant difference was found among both genders with respect to gastrointestinal problems. Respondent's age, educational status, work status, marital status, and morbidities, such as diabetes, stroke, and arthritis, were found to be the significant predictors of GI in our population. Moreover, difficulty in ADL, IADL, and other lifestyle factors, such as moderate and vigorous physical activity, smoking alcohol and consuming tobacco, and other household factors, such as wealth status, religion, caste, place of residence, and having non-communicable diseases, such as hypertension, heart disease, and neurological disorders were also found to be significantly associated with GI symptoms in Indian adults.

Globally, studies have reported varying rates of gastrointestinal problems, ranging from 14% in Iran to 54% in some western countries. Compare with other studies, GI prevalence is higher than in many neighboring nations and lower than that in many industrialized nations, such as the United States and Russia (17, 18). Many studies across the world have yielded different results about the prevalence of gastrointestinal problems and it has been reported to range from 14% in Iran to 54% in some western countries. The estimates obtained in our population are lower than the estimates reported in Sikkim and Darjeeling where 60% and 70% of people, respectively, reported complaining of gastrointestinal disease (15). The high prevalence of GI in some parts of the country may be due to the diet and cultural behaviors of the people (19). Moreover, these differences are also due to different study populations, lifestyles, and different diagnostic criteria used to estimate the prevalence.

Results from logistic regression analysis indicated that with the increase in age up to 64 years, the risk of having gastrointestinal problems increased significantly. However, this pattern was not significant at higher ages. Similar pieces of evidence have been found in a study conducted in India (19, 20).

In line with several western countries and some Indian studies, our findings showed that women were at a higher risk of gastrointestinal problems (20–22). Though the reasons are largely unknown, differences in access to healthcare and cultural factors, such as health-seeking behavior, may contribute to prevalence differences as well. However, a number of Indian studies have observed no significant gender differences in the risk of having gastrointestinal problems (23, 24). Further, higher education in adults was associated with a lower risk of gastrointestinal problems as found in a number of other studies worldwide. The increased awareness toward a healthy diet might be the possible explanation for the observed variation. The results on socio-economic status, wealth, and education are in line with findings across the world (25, 26).

Our findings indicated a strong association between having diabetes, arthritis, and difficulty in ADL and IADL activities and GI problems. Previous studies have documented the association between GI symptoms in patients with diabetes mellitus (27). We observed a high prevalence of gastrointestinal problems among those with psychiatric or neurological problems, which supported the other studies where psychological distress was found to be positively related to gastrointestinal problems (28). However, an in-depth examination of psychological distress and gastrointestinal problems shows that there exists a bidirectional relationship between stress and inflammatory bowel disease (IBD) symptoms. Moreover, patients with chronic health conditions, such as IBD, have a higher level of stress which impacts IBD (29, 30). Multiple lifestyle-related factors were shown to be related to inflammatory bowel syndrome, such as poor socioeconomic status, smoking, alcohol, and difficulty in ADL and IADL activities. In line with the already existing association, we found that vigorous activity significantly reduced the risk of having gastrointestinal problems (31, 32).

Additionally, smoking has been documented to be associated with an increased risk of inflammatory bowel disease (33). This study observed that individuals who quit smoking were more likely to have gastrointestinal problems, which is similar to the findings obtained by Cosnes and colleagues (34). According to our findings, despite the fact that this study suggests that alcohol users are more likely to experience gastrointestinal issues, alcohol has not consistently been linked to an increased risk of IBD development in other studies (35).

The rural-urban variation in the prevalence of this disease may be due to environmental and socio-demographic factors. Moreover, the sufficient intake of fruits and vegetables in their diet might be the possible reason behind the reduced risk of urbanites having gastrointestinal problems. The effect of dietary patterns on functional gastrointestinal disease has been well-established (28, 36). The regional variation in the prevalence of gastrointestinal problems can be attributable to cultural differences, ethnic diversity, genetics, and dietary habits. Current research uses data from the nationally representative study to show the importance of age, level of education, the influence of other illnesses, and substance abuse on gastrointestinal problems. Aging-related gastrointestinal problems are physiological or pathological and more prevalent in the population aged 64 years and above. The gastrointestinal system plays an essential role in medication absorption and metabolism. The GI system is frequently impacted by medication side effects and plays a crucial role in both drug absorption and metabolism. The management of GI disease among older adults, including prompt diagnostic and therapeutic methods, is complicated by the existence of complex comorbidities, lengthy drug intake, and other factors. GI problems among older adults are acquiring greater importance in clinical practices to plan effective treatment, administration of gastrointestinal drugs early screening, and management of GI diseases. Given the policy focus through Health and Wellness centers for accessible NCD care, it is important that gastro-intestinal illnesses receive more focus and systemic support. In the present study, the disease states under the survey are self-reported, with a potential for several recall/social-desirability biases, which might lead to gross under-reporting due to stigma or other forms of respondent behaviors. Moreover, the LASI survey only captures the self-reported prevalence of diagnosed gastrointestinal diseases. Additionally, there is no comprehensive report of the burden of GI diseases in India. To improve the quality of life of the older population, more population-level research is required to understand the types of GI diseases, as well as lifestyle, behavioral, and dietary factors that contribute to GI disorders.

## Data availability statement

The study used secondary datasets available in the public domain. These can be accessed upon reasonable request via https://www.iipsindia.ac.in/content/lasi-wave-i.

## Ethics statement

The studies involving human participants were reviewed and approved by the Indian Council of Medical Research (ICMR) extended the necessary guidelines and ethics approval for undertaking the LASI survey. The patients/participants provided their written informed consent to participate in this study.

## Author contributions

SD, PD, and ID conceived and designed the research paper and contributed agents, materials, and analysis tools. PD and SD analyzed the data and wrote the manuscript. ID refined the manuscript. All authors read, reviewed, and approved the manuscript.

## Conflict of interest

The authors declare that the research was conducted in the absence of any commercial or financial relationships that could be construed as a potential conflict of interest.

## Publisher's note

All claims expressed in this article are solely those of the authors and do not necessarily represent those of their affiliated organizations, or those of the publisher, the editors and the reviewers. Any product that may be evaluated in this article, or claim that may be made by its manufacturer, is not guaranteed or endorsed by the publisher.
